# CCL24 Protects Renal Function by Controlling Inflammation in Podocytes

**DOI:** 10.1155/2021/8837825

**Published:** 2021-06-16

**Authors:** Youdi Wang, Xue Wu, Mengya Geng, Jiamin Ding, Kangjia Lv, Hui Du, Jiahui Ding, Wenjun Pei, Xin Hu, Jing Gu, Lizhuo Wang, Yao Zhang, Jialin Gao

**Affiliations:** ^1^Department of Endocrinology and Genetic Metabolism, The First Affiliated Hospital of Wannan Medical College (Yijishan Hospital of Wannan Medical College), Wuhu 241002, China; ^2^Anhui Province Key Laboratory of Biological Macro-molecules Research, Wannan Medical College, Wuhu 241001, China; ^3^School of Clinical Medicine, Wannan Medical College, Wuhu 241002, China; ^4^Department of Biochemistry and Molecular Biology, Wannan Medical College, Wuhu 241001, China; ^5^Institute of Endocrine and Metabolic Diseases, Yijishan Hospital of Wannan Medical College, Wuhu 241002, China; ^6^Anhui Clinical Research Center for Diabetic Nephropathy, 2 Western Zheshan Road, Wuhu, Anhui 241001, China; ^7^Key Laboratory of Non-coding RNA Transformation Research of Anhui Higher Education Institution, Wannan Medical College, Wuhu 241001, China

## Abstract

Diabetic nephropathy (DN) is one of the most lethal complications of diabetes mellitus with chronic inflammation. We have examined the role of the inflammatory chemokine CCL24 in DN. We observed that serum levels of CCL24 were significantly elevated in patients with DN. Not only that, the expression of CCL24 was significantly increased in the kidneys of DN mice. The kidney of DN mice showed increased renal fibrosis and inflammation. We characterized an in vitro podocyte cell model with high glucose. Western blot analysis showed that expression of CCL24 was significantly increased under high-glucose conditions. Stimulation with high glucose (35 mmol/L) resulted in an increase in CCL24 expression in the first 48 hours but changed little after 72 hours. Moreover, with glucose stimulation, the level of podocyte fibrosis gradually increased, the expression of the proinflammatory cytokine IL-1*β* was upregulated, and the expression of the glucose transporter GLUT4, involved in the insulin signal regulation pathway, also increased. It is suggested that CCL24 is involved in the pathogenesis of DN. In order to study the specific role of CCL24 in this process, we used the CRISPR-Cas9 technique to knock out CCL24 expression in podocytes. Compared with the control group, the podocyte inflammatory response induced by high glucose after CCL24 knockout was significantly increased. These results suggest that CCL24 plays a role in the development of early DN by exerting an anti-inflammatory effect, at least, in podocytes.

## 1. Introduction

The prevalence of diabetes is rising rapidly around the world, and some studies predict that the number of diabetes patients worldwide will reach 592 million by 2035 [1]. The incidence rate of diabetic nephropathy (DN) is nearly 20-40%, in diabetic patients over 10 years [2]. The DN has become the major cause of end-stage renal disease (ESRD) in the western world and some developing countries [3], and it is difficult to cure, and also even more difficult to reverse when entering the ESRD. Therefore, effective prevention and treatment of diabetic nephropathy, even in early and mid-stages (definition as eGFR > 45 mL/min/1.73 m^2^), is of great significance in the treatment of DN.

At present, it is generally believed that, in addition to complex genetic factors, the onset of DN is also related to the activation of the polyol pathway [4]: the deposition of advanced glycation end products, the increased level of oxidative stress in the body, the renin-angiotensin-aldosterone system and protein, etc. In these pathogeneses, the importance of inflammation is gradually recognized [5]. Studies have confirmed that DN is in a “microinflammatory” state for a long time. This “microinflammation” is different from our common inflammatory diseases (such as furuncle and carbuncle) whose main clinical manifestations are redness, swelling, heat, and pain. The “microinflammation” of DN is essentially immune inflammation. The high-glucose environment associated with DN triggers a cascade of inflammation in the body, which affects the development of DN through macrophage infiltration and overexpression of inflammatory factors [6]. Under the condition of kidney tissue inflammation in diabetic nephropathy, the secretion of inflammatory factors (such as chemokines and adhesion factors) increases, and immune cells (macrophages, T cells, etc.) are recruited to accumulate and infiltrate the kidney tissue, leading to further release of proinflammatory factors, thereby aggravating inflammation and damaging the kidneys [7].

C-C motif chemokine ligand 24 (CCL24), also known as eotaxin2, belongs to CC chemokines like MCP-1, and it mainly chemoattracts eosinophils [8]. CCL24 can promote the separation of eosinophils from endothelial cells and then combine with its sole ligand to promote eosinophils to enter the tissue [9]. CCL24 was not originally found in eosinophils but was first isolated from monocytes. Latest, it was found that the expression of CCL24 in the bronchial epithelial cells of asthma patients increased [10]. Inhibiting the expression of CCL24 can reduce airway inflammation [11]. In addition, macrophages can also selectively induce the production of CCL24 [12]. However, there are few studies on CCL24 in the kidneys [13]. The specific role and mechanism of CCL24 in diabetic nephropathy are still unknown. In our early work, the human inflammatory antibody chip is used to screen the serum samples in patients with DN, and the CCL24 was found increased significantly in patients with early stages of DN. So we sought to explore the relationship between CCL24 and the development of early DN, which could potentially provide new therapeutic targets for the prevention and treatment of early DN.

## 2. Materials and Methods

### 2.1. Animal

SPF mice, half male and half male, were purchased from the Qinglongshan Animal Farm in Nanjing, China. After 3 days of adaptive feeding, the experimental mice were fed a high-fat and high-sugar diet (made in the laboratory. Formula: lard : sucrose : egg yolk : basic diet = 18 : 20 : 3 : 59) for 8 consecutive weeks, and starting from the first day of the experiment, STZ sodium citrate solution was injected at a dose of 100 mg/kg for 7 consecutive days [14]. On the 8th day, the blood glucose level of the mice was detected by the tail vein, and the blood glucose greater than 16.7 mmol/L was used as the model of diabetes model. DN mice need to have the following conditions: random blood glucose greater than 13.8 mmol/L, and positive urine protein is considered a successful model establishment.

### 2.2. Cell Culture

Mouse glomerular podocytes (MPC5, Cat. No. 337685) were purchased from Beijing Bei Na Chuanglian Biotechnology Research Institute. The cells were propagated in a medium recommended by the cell bank, which contained DMEM medium (GIBCO, Cat. No.12800017, added NaHCO3 1.5 g/L), 66.25%; F-12 medium (GIBCO, Cat. No. 21700075, added L-glutamine 150 mg/L, NaHCO3 1.5 g/L), 23.75%; 14 mM HEPES; and fetal calf serum (Gibco, USA), 10%. Standard culture conditions were maintained (gas phase: air, 95%; carbon dioxide, 5%; and temperature, 37°C).

### 2.3. Human Serum Specimen

Patients diagnosed with diabetes mellitus, with (*n* = 10) or without (*n* = 10) early diabetic nephropathy, were enrolled. Among them, the diagnostic criteria of early and mid-stage diabetic nephropathy are as follows [15]: (1) massive albuminuria, (2) within 6 months, the ratio of microalbuminuria/creatinine in urine was higher than 30 mg/g twice, (3) patients with diabetic retinopathy with trace albumin, (4) the course of microalbuminuria in patients with type 1 diabetes was >10 years, and (5) normal urinary creatinine. The above two groups of patients meet the diagnostic criteria of diabetes issued by the World Health Organization (WHO), and the following conditions are excluded: (1) strenuous exercise within 24 hours, (2) fever, (3) congestive heart failure, (4) pregnant women, (5) obvious hypertension, (6) upper respiratory tract infection, urinary tract infection, and other infections, (7) patients with tumors, rheumatoid diseases, and kidney diseases, and (8) recent use of hormone drugs, or other drugs that cause kidney damage. All participants were from the southern Anhui region, and all signed informed consent.

### 2.4. Gene Knockout Cell Line Construction

The mouse podocyte line stably knocking out CCL24 was constructed by the CRISP/Cas9 system [16], and the sgRNA (CACCGGCTCTGCTACGATCGTTGC) containing BbsI restriction site was designed. After annealing, double-stranded DNA was formed, and px459 was linearized with restriction endonucleases BbsI and sgRNA. After ligation, the ligated products were successfully transfected into mouse podocytes after electroporation. After 48 hours of culture, puromycin was added for screening, and the Western blot technique was used to determine the successful knockdown of CCL24, thereby obtaining CCL24 knockout mouse podocytes system.

### 2.5. Characterization of Pathological Changes in Mouse Glomeruli

The normal diet group and the HFD/STZ diet group were set up. HE (hematoxylin-eosin) staining, PAS (periodic acid-Schiff) staining, Masson's trichrome staining, and immunofluorescence staining were used to observe the glomerular pathological changes and immune complex deposition in the two groups.

### 2.6. Changes of Mouse Glomerular Nidogen Expression

Immunofluorescence staining was used to observe changes in glomerular basement membrane in DN mice. Renal tissue sections were fixed in 4% paraformaldehyde, after washing 3 times with PBS, blocked with 3% BSA for 30 min, and incubated with Nidogen (Abcam, Cat. No. ab 131279) primary antibody (1 : 500) at 4°C overnight. The next day, they were incubated with the anti-rabbit fluorescent secondary antibody (1 : 200) for 2 h at room temperature and washed with PBS to analyze under inverted fluorescence microscope (Olympus) (for details, please see the previous publications [17]).

### 2.7. High-Glucose Stimulation Testing MPC5 Cell Lines

After 2 weeks of culture, podocytes with good growth status were transferred to a six-well plate. Cells were cultured in 6-well culture dishes until they reached approximately 50% confluence and were then stimulated with glucose in different concentrations and time. The concentration gradient and time gradient were set separately. There were five groups, including three high-glucose concentration gradients, a normal glucose concentration control, and a hypertonic control. Concentration gradient is as follows: Well 1 was set as the normal control group (D-glucose 5.5 mmol/L), well 2 as the hyperosmotic group (D-glucose 5.5 mmol + mannitol 39.5 mmol), well 3 as a high glucose group with D-glucose 25 mmol/L, well 4 as a high glucose group with D-glucose 35 mmol/L, and well 5 as a group with D-glucose 45 mmol/L. After that, the residual medium was removed and washed three times with cold PBS buffer, and the cells were scraped off on ice. Finally, total protein was extracted, and the expression of CCL24 and the key proteins such as inflammatory signaling pathway and glucose metabolism pathway were detected by Western blotting.

### 2.8. Western Blot Analysis

For Western blot analysis, several primary antibodies were used. Signals were detected using HRP-conjugated secondary antibodies and the ECL detection reagent (Beyotime, China). Details can be found in our previous study [18]. Primary antibodies used in the present study include CCL24 (ProteinTech, 22306-1-AP), podocin (Sigma, P0372), Fibronectin-1 (CST, 63779S), IL-1*β* (ProteinTech, 66737-1-Ig), GLUT4 (CST, 2213S), IKK*β* (Beyotime, China, AF7200), and *β*-actin (Sigma, A5441).

### 2.9. Statistical Analysis

Results of the data analysis are presented as mean ± SEM. Statistical comparison between two groups was performed using unpaired *t*-tests. Statistical analyses were performed using the GraphPad prism 6.0 software, and *p* < 0.05 was considered statistically significant.

## 3. Results

### 3.1. Comparison of Clinical Indexes between Diabetic and Early DN Patients

There was no significant difference in gender composition and age between diabetic patients (*n* = 10) and early DN patients (*n* = 10) included in the study. In the early DN patients included in the study, urine microalbumin, urine microalbumin/urinary creatinine, 24 h urine protein quantification, creatinine, and cystatin-C were all higher than those of diabetic patients, but the eGFR was reduced, and they were statistically significant (*p* < 0.05). There was no significant difference in glycemia (fasting plasma glucose and HbA1c), HDL, and LDL between the two groups (Table 1).

### 3.2. Higher Levels of CCL24 in the Serum of Patients with Early DN

**Figure 1:**
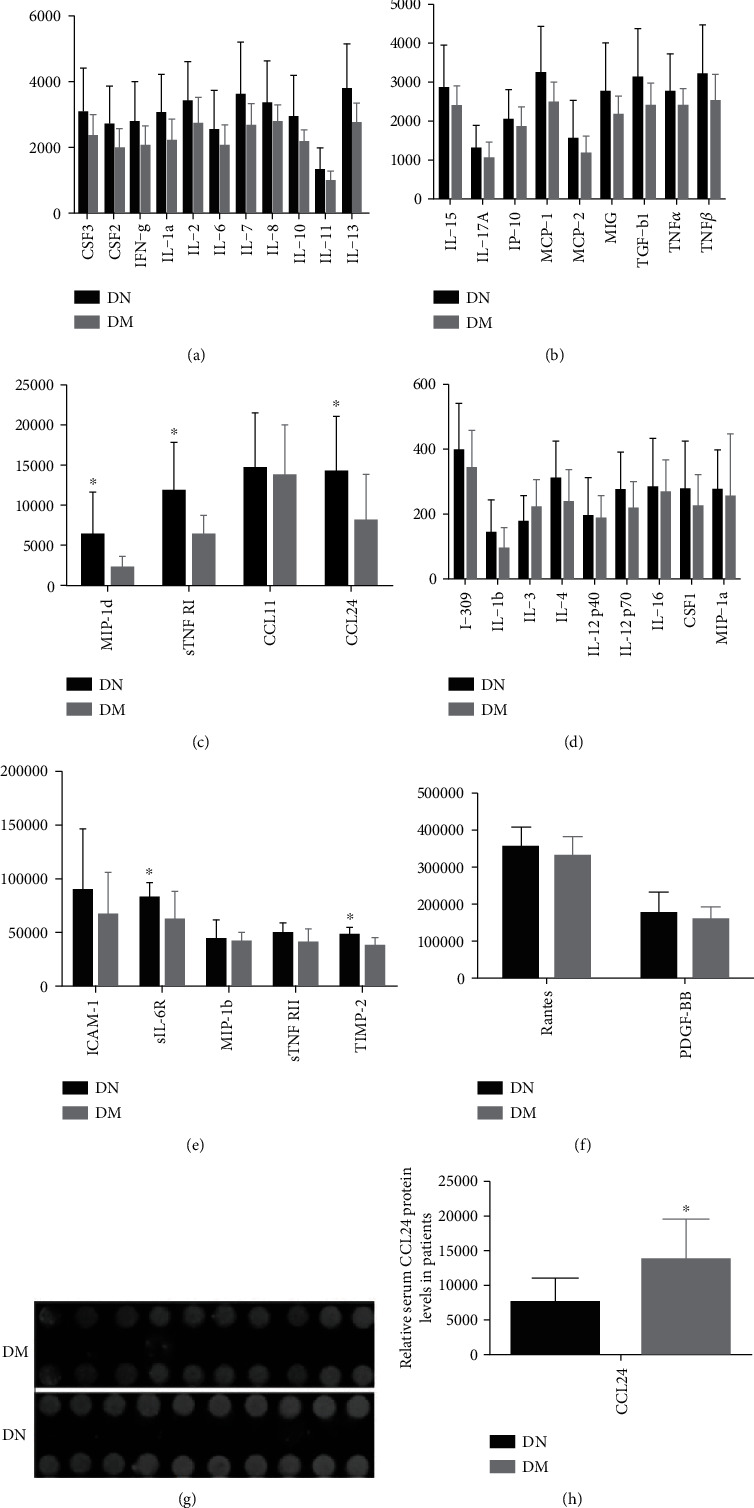
The serum CCL24 in patients with early DN is significantly increased compared with diabetic patients. (a–f) Serum inflammation antibody chip screening results in patients with diabetes and early DN. (g, h) Fluorescence intensity and statistical results of serum CCL24 inflammatory antibody chip in patients with diabetes mellitus and early and DN. *n* ≥ 3, ^∗^*p* < 0.05, ^∗∗^*p* < 0.01, ^∗∗∗^*p* < 0.001. DN: patients with diabetic nephropathy, DM: diabetes patients without DN.

By the microarray chip analysis, the results showed that in early DN patients, 40 inflammatory factors, including IL-1*β*, IL-6, and TNF*α*, were elevated compared with those without DN, and the differences in six inflammatory factors, including CCL24, were statistically significant (Figures 1(a)–1(f)). The fluorescence intensity of CCL24 in patients with early DN was significantly higher than that in those without DN, and the difference between them was statistically significant (Figures 1(g) and 1(h)).

### 3.3. Successfully Constructed Mouse Model of DN

**Figure 2:**
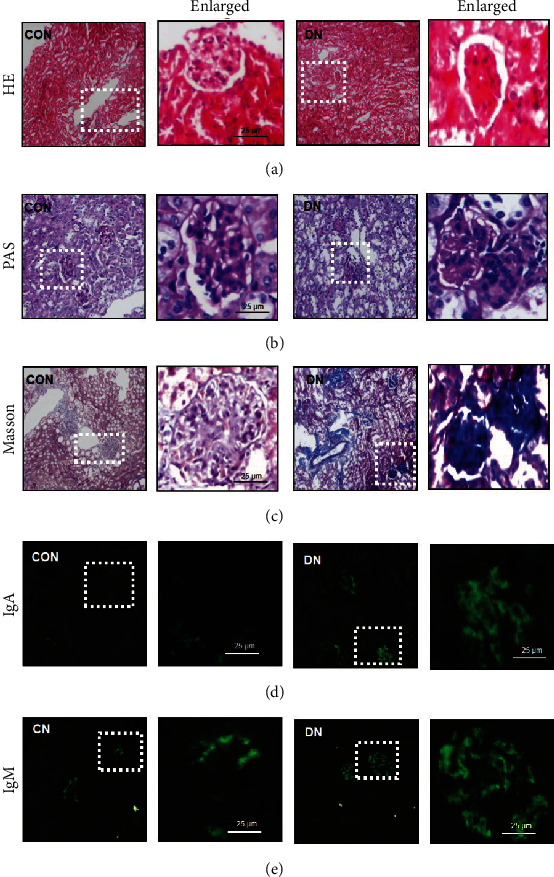
Renal section staining results. (a) HE staining, (b) PAS staining, (c) Masson's staining, and immunofluorescence staining against (d) IgG and (e) IgM in control and DN mice. CON: control mice, DN: diabetic nephropathy mice.

On the basis of the early detection of CCL24 in the serum of patients with DN, we reasoned that CCL24 might be involved in the development of DN. Therefore, we used the classic model involving STZ treatment combined with high-fat feeding to generate a mouse model of DN. We found that the fasting blood glucose, fasting HbA1c, total cholesterol, HDL-C, LDL-C, and renal index were higher in the DN mice than in control mice. Moreover, the plasma albumin and body weight of the mice with DN were significantly lower than those of control mice (Table 2). Next, we performed morphological analysis of the kidneys from DN mice. HE staining revealed diffuse hyperplasia of mesangial cells and glomerular atrophy in the DN mice compared with control mice (Figure 2(a)). PAS staining showed glomerular basement membrane wrinkles and thickening, tubular basement membrane thickening, and mesangial cell hyperplasia in the DN mice compared with control mice (Figure 2(b)). The Masson staining showed significantly more renal fibrosis in DN mice than the control mice (Figure 2(c)). In addition, there were obvious granular deposition of IgA (Figure 2(d)) and IgM (Figure 2(e)) in the mesangial cells of DN mice, and it is also one of the atypical pathological manifestations of diabetic nephropathy (Figures 2(d) and 2(e)).

### 3.4. Renal Dysfunction and Increased Expression of CCL24 in DN Mice

**Figure 3:**
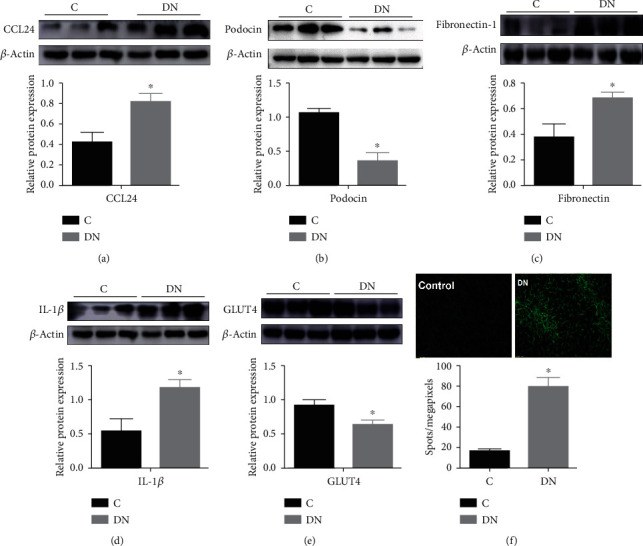
Molecular changes in kidney from DN mice. Western blot analysis of (a) CCL24, (b) podocin, (c) Fibronectin-1, (d) IL-1*β*, and (e) GLUT4. (f) Immunofluorescence analysis of Nidogen 2 in the kidney from control and DN mice. C: control mice, DN: diabetic nephropathy mice.

At the molecular level, we also examined the expression of CCL24 in the kidney of DN mice by Western blotting and found that the expression of CCL24 was enhanced compared with that in control mice (Figure 3(a)). In addition, the expression of podocin, a protein located to the podocyte membrane, was lower in the DN mice (Figure 3(b)), thus, suggesting podocyte damage. The degree of renal fibrosis, assessed through expression of Fibronectin-1 (Figure 3(c)), and the expression of proinflammatory IL-1*β* (Figure 3(d)) were greater, and the expression of glucose transporter GLUT4 was lower (Figure 3(e)) in DN compared to control mice. Using immunofluorescence, we also found that the expression of Nidogen in the kidney of DN mice was significantly elevated, thus suggesting the presence of glomerular basement membrane thickening (Figure 3(f)).

### 3.5. CCL24 Is Involved in Renal Fibrosis and Inflammation in Mice with DN

**Figure 4:**
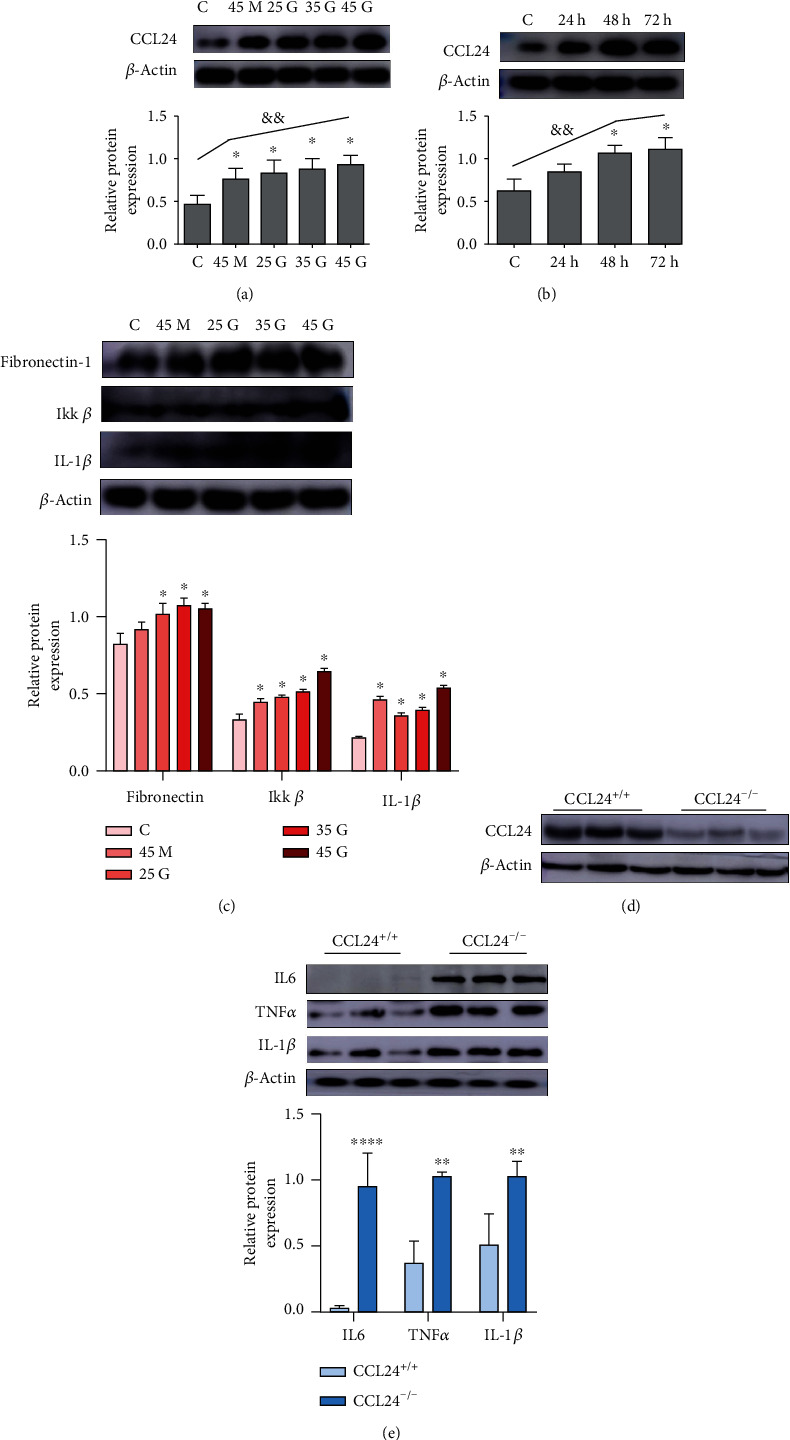
CCL24 is a potential protective factor for diabetic nephropathy by controlling inflammation in podocytes. The expression of CCL24 was positively correlated with (a) glucose concentration and (b) the duration of stimulation. (c) The level of fibrosis and the regulation of inflammatory regulatory pathways in the podocytes treated with high-glucose stimulation. (d) Molecular level detection after knockout of CCL24 gene by the CRISP-Cas 9 technology at podocyte level. (e) The changes of inflammatory pathways in CCL24^−/−^ cell lines under high-glucose conditions (35 mmol/L D-glucose). C: the control of 5.5 mmol/L D-glucose, 45 M: 5.5 mmol glucose + mannitol 39.5 mmol, 25G: 25 mmol/L D-glucose, 35G: 35 mmol/L D-glucose, 45G: 45 mmol/L D-glucose. All data are presented as mean ± SEM. ^∗^*p* < 0.05, ^∗∗^*p* < 0.01, and ^∗∗∗∗^*p* < 0.0001 versus the control group (C) or CCL24^+/+^ group. ^&&^*p* < 0.001, the time- and concentration-dependent variance trend test.

At the cellular level, we used a high-glucose podocyte model to assess CCL24 expression. The expression of CCL24 was significantly higher in the hypertonic and high-glucose state than that in the control, as determined by Western blotting (Figure 4(a)), and the expression of CCL24 gradually increased with the increase of glucose concentration (Figure 4(a), ^&&^*p* = 0.003, variance trend test) and duration of action, respectively (Figure 4(b), ^&&^*p* = 0.006, variance trend test). In addition, stimulation with high glucose showed an increase in the overall level of inflammation, which was manifested as an increase in the expression of IKK*β* and IL-1*β* in podocytes (Figure 4(c)). Moreover, the expression of Fibronectin-1 in the podocytes increased, thus suggesting epithelial to mesenchymal transition. From these results, we speculated that the high-glucose environment might induce upregulation of CCL24 expression in podocytes, thus increasing inflammation. To investigate this possibility, we used the CRISPR-Cas9 technology to obtain CCL24^−/−^ podocytes. The expression of CCL24 in the CCL24^−/−^ group was nearly absent and significantly lower than that in the control group (Figure 4(d)). Subsequently, compared with the control group, we found that the expression of IL-6, IL-1*β*, and TNF*α* was significantly increased in the knockout group, which indicated the inflammatory pathway was activated (Figure 4(e)).

## 4. Discussion

CCL24 can promote the separation of eosinophils from endothelial cells by downregulating the inflammatory factor VCAM-1 and then combine with its sole ligand CCR3 to promote eosinophils to enter the tissue [19]. While CCL24 regulates inflammatory factors, it is also regulated by other inflammatory factors. For example, TNF*α*, IFN*γ*, and IL4 can stimulate the expression of CCL24 in a dose-dependent manner [20]. There are few studies on CCL24 in the kidney diseases. Here, we used inflammatory antibody microarrays to screen serum from patients with diabetes and early DN, and we found that CCL24 expression was increased significantly in patients with DN. We speculate whether the inflammatory chemokine CCL24 plays an important role in the occurrence and development of DN. Therefore, we used HFD/STZ to induce a DN model in C57BL/6 mice at first [14, 21], and the expression of CCL24 in the kidney of DN mice was significantly higher than that in control.

Furthermore, by Western blotting analysis, we also found that the expression of podocin in the kidney in DN mice was significantly lower than that in control mice. Podocin is a key protein on the pores of podocyte cells. The downregulation of podocin expression suggests that the podocytes are damaged in DN [22]. The main laboratory markers of DN progression are albuminuria and a reduction in glomerular filtration rates, and podocytes play a major role in regulating glomerular filtration. This podocyte damage is the earliest change, which leads to the destruction of the glomerular filtration membrane and to proteinuria [23, 24]. Expression of Fibronectin-1 in the kidney in the DN mice was significantly elevated, and the inflammatory factor IL-1*β* increased, thus suggesting an increase in renal fibrosis and inflammation in the DN mice model. Moreover, the glucose transport function in the DN model may also have been impaired, owing to the downregulation of GLUT4 expression. This series of pathological changes in DN model prompted us to explore the related role of CCL24 playing in the development of DN.

Podocytes are an important component of the glomerular filtration barrier and are suggested to be the first to develop lesions in DN [22]. Therefore, we explored the role of CCL24 in normal mouse podocytes. Under the pathological conditions of DN, glucose is the main stimulating factor [14]. So, we stimulated podocytes with a glucose concentration gradient. The results showed that CCL24 protein levels were positively correlated with blood glucose concentration and glucose action time. The level of fibrosis gradually increased and was accompanied by an increase in proinflammatory factors, thus suggesting that CCL24 may play a vital role in regulating podocyte function. Further results have also indicated an increase in IL-1*β* which was upstream mediator of the MAPK pathway. Accompanied with the increase of CCL24, the NF*κ*B signaling pathways were also activated. In addition, high-glucose-stimulated podocyte insulin signaling pathways were also abnormal. So whether there is a correlation between the abnormal insulin signaling pathways and the upregulation of CCL24 expression as well as the activation of inflammatory pathways needs further investigation [25].

Many inflammatory chemokines are currently found to be correlated to the pathogenesis of DN [26]. Usually, most inflammatory factors act as pathogenic factors, and their expression levels are positively correlated with the progression of diabetic nephropathy [27]. For example, hyperglycemia can stimulate the secretion of CCL2, CCL5, and CXCL12, through podocytes and tubular cells, as reported in mouse models and DN patients, which contributes to proteinuria and glomerulosclerosis [28–30]. However, in a few cases, some inflammatory factors exist and act as protective factors, such as interleukin 17A (IL-17A). IL-17A serum levels were increased significantly in experimental DN model of T1DM [31], and kidney injury is more severe in IL-17A knockout mice with STZ-induced diabetes. More interesting, the administration of recombinant IL-17A to a mice model for T1DM reduced albuminuria and renal injury [32]. So what is the real role of CCL24? Next, we used the CRISP-Cas9 technology to knock out CCL24 expression to study the effect of CCL24 deletion on podocyte inflammation. After CCL24 knock out, the podocyte inflammatory level increased significantly, compared with the control. The expression of IL-1*β*, IL-6, and TNF*α* was upregulated, and the NF*κ*B signaling pathway was also activated, which indicated that CCL24 played an inhibitory role in the inflammatory pathway. So far, we believe that in DN, CCL24 is closely related to renal inflammation, which can protect the renal function of patients with early DN by controlling inflammation. However, the specific mechanism of CCL24 is still unclear, and that will be addressed in further research, which could be a new target for the diagnosis and treatment of the DN.

## Figures and Tables

**Table 1:** Comparison of clinical indicators between diabetic patients without DN and with early DN.

	Diabetes without DN (*n* = 10)	Diabetes with early DN (*n* = 10)	*p*
*n* (male/female)	5/5	6/4	0.500
Age (year)	62.13 ± 8.69	61.75 ± 8.05	0.930
Urinary microalbumin (mg/L)	6.37 ± 5.87	459.18 ± 219.06	0.001^∗^
Urinary microalbuminuria/urinary creatinine (mg/g)	6.98 ± 5.67	918.17 ± 680.46	0.002^∗^
24 h urine protein quantitation (g)	0.04 ± 0.03	1.26 ± 1.01	0.004^∗^
Creatinine (*μ*mol/L)	59.17 ± 15.82	92.38 ± 19.39	0.001^∗^
eGFR	97.29 ± 16.74	62.31 ± 18.82	0.001^∗^
Cystatin-C (mg/L)	1.03 ± 0.28	1.59 ± 0.49	0.006^∗^
High-density lipoprotein (mmol/L)	1.23 ± 0.13	1.25 ± 0.38	0.727
Low-density lipoprotein (mmol/L)	2.40 ± 0.52	2.89 ± 0.77	0.72
Fasting plasma sugar (mmol/L)	7.22 ± 3.82	7.65 ± 4.18	0.15
HbA1c (%)	7.30 ± 1.80	7.10 ± 2.10	0.26

**Table 2:** Comparison of physiological parameters between control mice and DN mice.

	Control	DN
Fasting blood sugar (mmol/L)	5.80 ± 2.81	27.25.37 ± 6.6^∗^
HbA1c (%)	2.72 ± 0.41	7.00 ± 1.29^∗^
Creatinine (*μ*mol/L)	1089.9 ± 748.36	2030 ± 1037.82
Urinary microalbumin (mg/L)	13.95 ± 10.56	19.75 ± 10.75
Plasma albumin (g/L)	26.24 ± 1.38	20.34 ± 1.47∗
TG (g/L)	0.96 ± 0.40	1.054 ± 0.23
TC (mmol/L)	2.44 ± 0.50	5.62 ± 1.22^∗^
High-density lipoprotein-C (mmol/L)	1.33 ± 0.32	4.47 ± 2.45^∗^
Low-density lipoprotein-C (mmol/L)	0.31 ± 0.10	0.57 ± 0.16^∗^
Kidney weight/weight (g/g)	0.01 ± 0.001	0.02 ± 0.006^∗^
Body weight (g)	42.94 ± 3.94	31.61 ± 3.66^∗^

## Data Availability

The datasets used and/or analyzed during the current study are available from the corresponding author on reasonable request.
